# Dietary Copper Intake and Risk of Stroke in Adults: A Case-Control Study Based on National Health and Nutrition Examination Survey 2013–2018

**DOI:** 10.3390/nu14030409

**Published:** 2022-01-18

**Authors:** Lixiang Yang, Xun Chen, Huilin Cheng, Lihua Zhang

**Affiliations:** 1 Department of Neurosurgery, Zhongda Hospital, School of Medicine, Southeast University, Nanjing 210009, China; yangneurovascular@126.com; 2 Department of Cognitive Neurolinguistics, School of Foregin Language, East China University of Science and Techonology, Shanghai 200237, China; xunxunshu@aliyun.com; 3 Department of Pathology, Zhongda Hospital, School of Medicine, Southeast University, Nanjing 210009, China

**Keywords:** dietary copper intake, stroke, health risk, propensity score matching, restricted cubic spline

## Abstract

The association between dietary copper intake and the risk of stroke is unknown. We included a total of 10,550 participants from the National Health and Nutrition Examination Survey (NHANES) 2013–2018. Two 24-h dietary recalls and a standard questionnaire were used to determine copper intake and stroke, respectively. We used logistic regression models to estimate the associations between dietary copper intake and the risk of stroke. The nearest-neighbor propensity score matching (PSM) with a ratio of 1:2 was used to reduce selection bias. The non-linear relationship was explored with restricted cubic splines (RCS). The correlation between copper intake and baseline characteristics was detected by the Pearson correlation coefficient. The median dietary copper intake was 1.072 mg/day (IQR = 1.42–0.799). Approximately 3.8% (399) of the participants had a history of stroke. A multivariate logistic regression analysis before and after matching showed that subjects in the higher quartile had significantly lower odds of stroke compared with subjects in the first quartile of copper intake. A stratified analysis showed that copper intake was a significant protective factor for women, individuals <65 years old, individuals with hypertension, individuals who smoke, and diabetic stroke patients. The RCS models showed an L-shaped nonlinear relationship (*p* for nonlinear < 0.001) between copper intake and stroke. Our results suggested that increased dietary copper intake was associated with a lower risk of stroke.

## 1. Introduction

Stroke is currently the second leading cause of death in the world after ischemic heart disease [1]. On average, 86.5 per 100,000 people worldwide die of stroke every year [2]. The etiology and pathological process of stroke are complex, which can interfere with cerebral blood flow, thereby damaging normal brain tissues and causing central nervous system damage. Stroke survivors may be left with speech, cognitive, and strength dysfunction [3]. Although previous studies have shown that hypertension, diabetes, etc. are the main risk factors for stroke [4], it is also very necessary to identify new risk factors and provide information for the primary prevention of stroke. Recently, the role of the trace element copper in nervous system disease has received attention [5,6].

Copper is an essential element for the normal development and function of the human body. It cannot be manufactured or stored in the body and must be obtained from food and drinking water every day [7]. As a cofactor or structural component of several enzymes, copper is involved in many biological processes of the body, such as neurotransmitter synthesis (dopamine-β-monooxygenase), energy metabolism (cytochrome c oxidase), antioxidant defense (superoxide dismutase containing zinc and copper), etc. [8]. Studies have shown that when the homeostasis of copper was disturbed, it could lead to neurological impairment and neurological diseases, such as Wilson’s and Alzheimer’s diseases [9]. In previous studies, the relationship between serum copper concentration and cardiovascular disease was also explored. Bagheri et al. found that serum copper levels were elevated in patients with atherosclerosis and increased with the severity of atherosclerosis [10]. Some studies showed that the elevated plasma copper level was associated with an increased risk of stroke [11,12], while the serum copper level of patients with acute hemorrhagic stroke was significantly lower than that of the control group in another clinical study [13]. In addition, an animal experiment showed that chronic intake of copper might reduce angiogenesis and aggravate ischemic injury in mice [14]. In general, the relationship between copper and stroke risk remains controversial. Moreover, only a few studies focused on the relationship between dietary copper intake and stroke risk.

To fill the above research gap, we conducted a large case-control study to investigate the potential association between dietary copper intake and stroke risk based on the 2013–2018 NHANES data. This will also provide evidence for the prevention of stroke.

## 2. Methods

### 2.1. Data Source

The NHANES study is a multi-stage, stratified, large-scale, nationally representative study of the American population. It is conducted by the National Center for Health Statistics of the Centers for Disease Control and Prevention of the United States and aims to assess the nutritional and physical status of Americans [15]. The content of this cross-sectional survey includes demographic data, dietary data, examination data, questionnaire data, etc. A detailed description of the NHANES protocol has been published elsewhere [16]. The data of this study was obtained from the 2013–2018 NHANES database.

### 2.2. Copper Intake

All subjects were allowed to participate in two 24-h dietary recall interviews. The first recall interview was collected in an NHANES Mobile Examination Center (MEC), and the second dietary recall interview was conducted by telephone after 3–10 days. By using the U.S. Department of Agriculture’s Food and Nutrition Database for Dietary Studies, the daily total of all nutrients/food ingredients from all foods was calculated and entered into the NHANES database [17]. The average copper intake of the two 24-h recalls was used for analysis.

### 2.3. Definition of Disease and Covariates

As previously described, we determined stroke based on the Medical Condition Questionnaire (MCQ). When subjects answered “yes” to the question “Has a doctor or other health professional ever told you that you had a stroke?”, they were considered to have a stroke [18]. The determination of asthma and arthritis was similar to this. Subjects were considered to have diabetes if met one of the following conditions: people who had been told by doctors or other health professionals they have diabetes, people who were taking insulin, or people who were taking diabetic pills to lower their blood sugar [19]. The 9-item Patient Health Questionnaire (PHQ-9) depression scale was used to evaluate depression symptoms, and its total score was 0–27. Research showed that the sensitivity of a PHQ-9 total score ≥ 10 for major depression was 88%, and the specificity was 88% [20]. In this study, subjects with a PHQ-9 total score ≥ 10 were considered to have clinically relevant depression. Subjects who met one of the following conditions were considered to have hypertension: their mean systolic blood pressure (SBP) ≥ 140 mmHg or diastolic blood pressure (DBP) ≥ 90 mmHg in the examination data section, they had been informed of hypertension by doctors or other health professionals, they had been told to take a prescription for hypertension or they were taking a prescription for hypertension [21]. In the NHANES questionnaire data, the subjects listed the names and main reasons for taking prescription drugs in the past 30 days. After confirming that the prescription drug was a standard anti-epileptic drug, we defined the participant who answered at least one “epilepsy and recurrent seizure” (G40) drug as a patient with epilepsy [22]. We divided the smoking status into never smoking (<100 cigarettes in a lifetime) and current or ever smoking (≥100 cigarettes in a lifetime) [23].

According to previous studies, we also included the following covariates of interest: sex, age, race (Mexican American, other Hispanic, non-Hispanic white, non-Hispanic black, and other race), marital status (married/living with partner, widow/divorced/separated, and never married), family poverty income ratio (PIR, categorized as <1 and ≥1), education level (<less than 9th grade, 9–11th grade, high school graduate/GED or equivalent, some college or AA degree, and college graduate or above), insurance, high-density lipoprotein (HDL), general health (excellent, very good/good, and fair/poor), and trouble sleeping.

### 2.4. Ethics Statement

The NHANES study has been approved by the National Center for Health Statistics Research Ethics Review Board, and all participants in the survey signed informed consent. No ethical or administrative permission is required to access the NHANES database. More details are available online (www.cdc.gov/nchs/nhanes/ accessed on 11 December 2021).

### 2.5. Statistical Analysis

The extraction and merging of the 2013–2018 NHANES data were completed with R studio (version 4.1.1). All data analysis and figure design were completed by SPSS software (version 22.0), R studio (version 4.1.1), and GraphPad Prism (version 8.0). Continuous variables in baseline characteristics were analyzed by Student’s *t*-tests or nonparametric Mann–Whitney U tests, while categorical variables in baseline characteristics were analyzed by Chi-square tests or Fisher’s tests. Logistic regression analyses were used to analyze some possible risk factors for stroke. Propensity score matching was first proposed by Rubin and Rosenbaum in 1983 and has been widely used to reduce selection bias in observational studies [24,25]. It is based on counterfactual concepts and can help strengthen causal arguments in observational studies by reducing selection bias [26]. In this study, PSM, using the 1:2 nearest neighbor matching algorithm, was used to match participants with and without stroke. Confounding factors, including age, sex, race, education, pir, marital status, insurance, smoking, hypertension, HDL, asthma, arthritis, diabetes, general health, PHQ-9 depression score, trouble sleeping, and epilepsy, were chosen for matching. The PSM was conducted by R software. A stratified analysis was used to test whether the relationship between copper intake and stroke risk differed by sex, age, hypertension, smoking status, and diabetes. The restricted cubic spline function is a useful tool to describe dose-response relationships between continuous exposure and outcomes for a priori non-linear associations or to test the hypothesis of linearity before entering a continuous variable in a model with appropriate recoding [17,27]. The RCS function is also suggested to be used when adjusting for continuous exposures to minimize residual confounding [28,29]. In this study, we used an RCS model with four knots to further explore the association between copper intake and stroke risk, before and after adjusting for the confounders. The RCS function was performed by R software. In addition, the correlation between copper intake and baseline characteristics was detected by the Pearson correlation coefficient. *p* < 0.05 was considered statistically significant.

## 3. Results

### 3.1. Participant Characteristics

**Figure 1:**
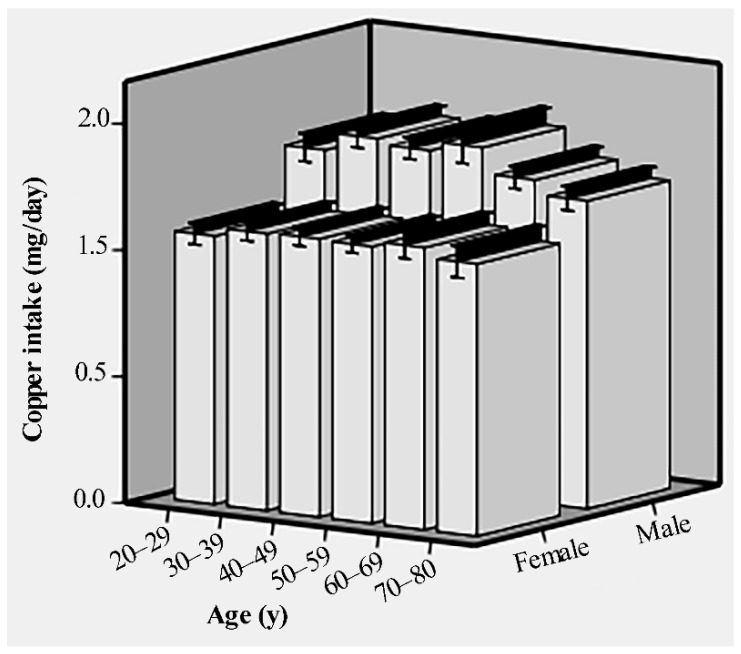
The dietary copper intake of males and females in different age groups before matching.

Of the 10,550 participants, the age range was 20–80 years, and the mean age was 50 ± 17.35 years. About 52.3% of the subjects consisted of females, 41.5% were non-Hispanic white, 32.5% had some college or AA degree education, and 60.7% were married/living with partner. The baseline characteristics of the subjects are provided in Table 1. Compared with subjects in quartile 1 and quartile 2 of copper intake, subjects in quartiles 3 and 4 were more likely to be male, younger adults, married/living with partner, PIR ≥ 1, and have a college graduate or above education. The copper intake of males and females in different age groups is shown in Figure 1.

### 3.2. Analysis of Risk Factors for Stroke before and after Matching

**Figure 2:**
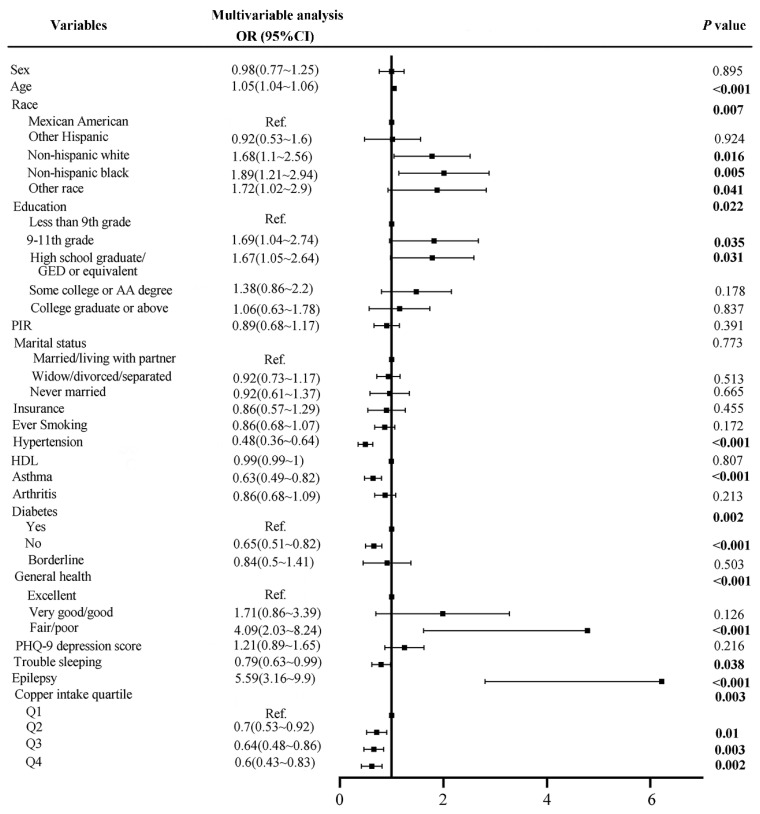
Forest plot of stroke risk factors before matching.

As shown in Table 2, there were 399 cases of stroke. All covariates were associated with stroke (*p* < 0.05), except for sex and HDL. The multivariate analysis (Figure 2) showed that the risk of stroke would increase by 1.05 times as the age increases by one year, *p* < 0.001. Subjects in non-Hispanic white, non-Hispanic black, and other race groups had a 1.68-, 1.89-, and 1.72-times higher risk of stroke than the Mexican American group, respectively, *p* < 0.05. The probability of stroke in subjects without hypertension was 0.48 times that of subjects with hypertension, *p* < 0.001. No history of asthma (OR = 0.63, *p* < 0.001), diabetes (OR = 0.65, *p* < 0.001), and trouble sleeping (OR = 0.79, *p* = 0.038) were all protective factors for stroke. However, fair/poor general health (OR = 4.09, *p* < 0.001) and epilepsy (OR = 5.59, *p* < 0.001) were risk factors for stroke. Most interestingly, the stroke risks of quartiles 2, 3, and 4 of dietary copper intake were 0.7 (*p* = 0.01), 0.64 (*p* = 0.003) and 0.6 (*p* = 0.002) times that of quartile 1, respectively.

**Figure 3:**
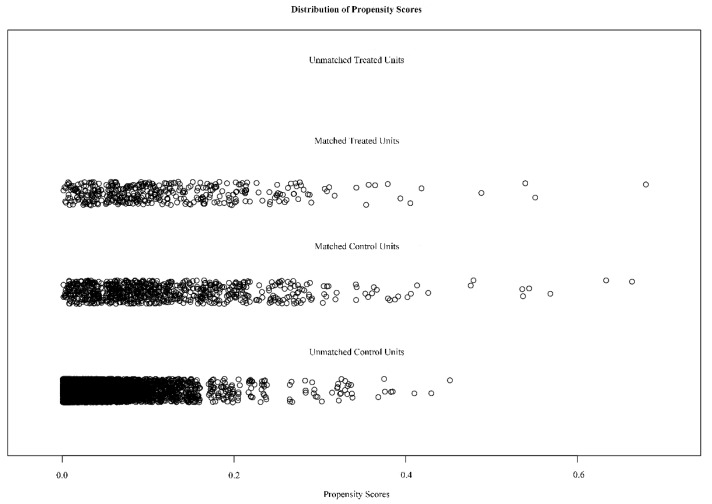
Distribution of propensity scores for the matched and unmatched treatment and control subjects with a ratio of 1:2.

**Figure 4:**
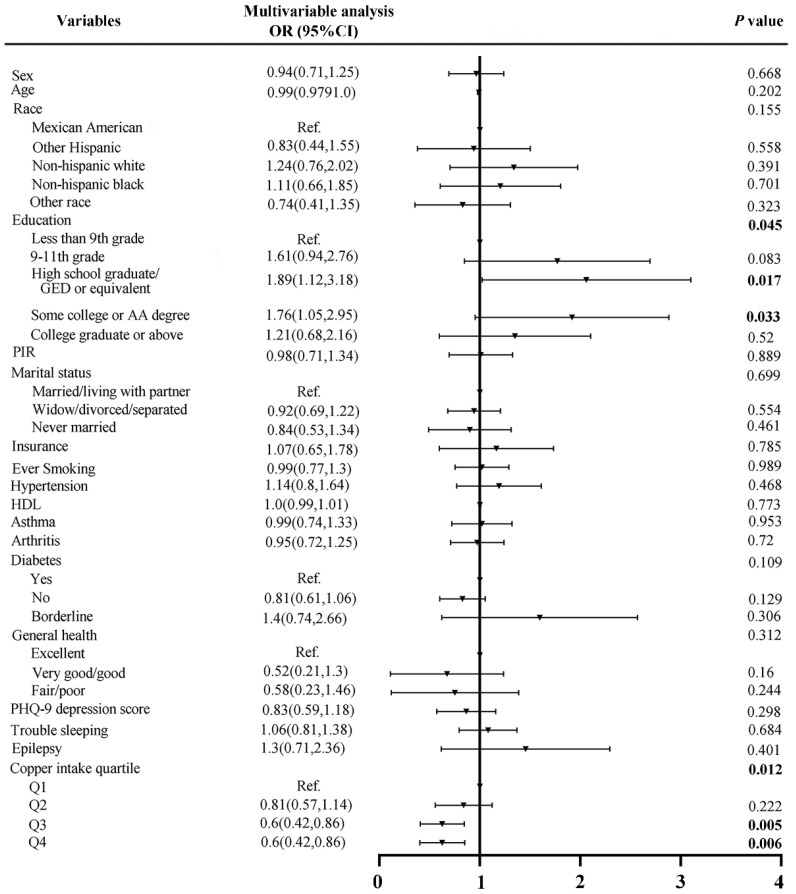
Forest plot of stroke risk factors after matching.

To further verify the association between dietary copper intake and the risk of stroke, we created a comparable control group using the nearest neighbor propensity score matching method (1:2) (Figure 3). After propensity score matching, we included 798 and 399 participants in the control group and stroke group, respectively. Subsequent analysis results (Table 2) showed that, except for race (*p* = 0.048) and education (*p* = 0.01), other baseline characteristics were not statistically significant between the control and stroke groups. Consistent with before matching, copper intake was still statistically significant (*p* = 0.007) between the two groups after matching. The results of the subsequent multivariate analysis showed that the stroke risks of quartiles 3 and 4 of copper intake were 0.6 (*p* = 0.005) and 0.6 (*p* = 0.006) times that of quartile 1, respectively (Figure 4).

Subsequently, a stratified analysis was used to assess whether the association between copper intake and stroke risk differed by sex, age (<65 and ≥65 years), smoking status, hypertension, and diabetes. When the analysis was stratified by sex, the association between copper intake and stroke risk was more significant in female individuals (*p* = 0.002; Table S1). Similarly, the association between copper intake and stroke was more meaningful in individuals aged <65 years old (*p* = 0.013; Table S2), individuals with hypertension (*p* = 0.002; Table S3), individuals who smoke (*p* = 0.027; Table S4), and individuals with diabetes (*p* = 0.013; Table S5).

### 3.3. Nonlinear Association

**Figure 5:**
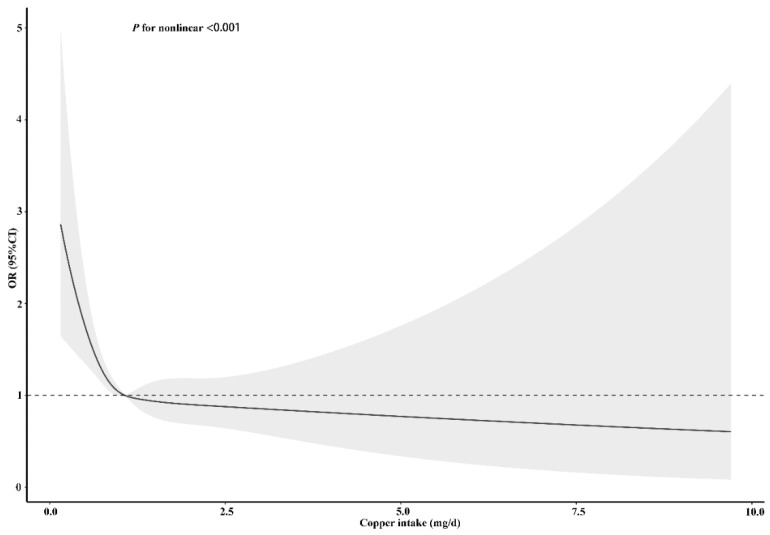
Restricted cubic spline models for the relationship between dietary copper intake and the risk of stroke before matching. The 95% CIs of the adjusted ORs are represented by the gray-shaded area. The model is adjusted for sex, age, race (Mexican American, other Hispanic, non-Hispanic white, non-Hispanic black, or other race), education (less than 9th grade, 9–11th grade, high school, graduate/GED or equivalent, some college or AA degree, or college graduate or above), PIR, marital status (married/living with partner, widow/divorced/separated, or never married), insurance, smoking, hypertension, HDL, asthma, arthritis, diabetes (yes, no, or borderline), general health (excellent, very good/good, or fair/poor), PHQ-9 depression score, trouble sleeping, and epilepsy. OR, odds ratio.

**Figure 6:**
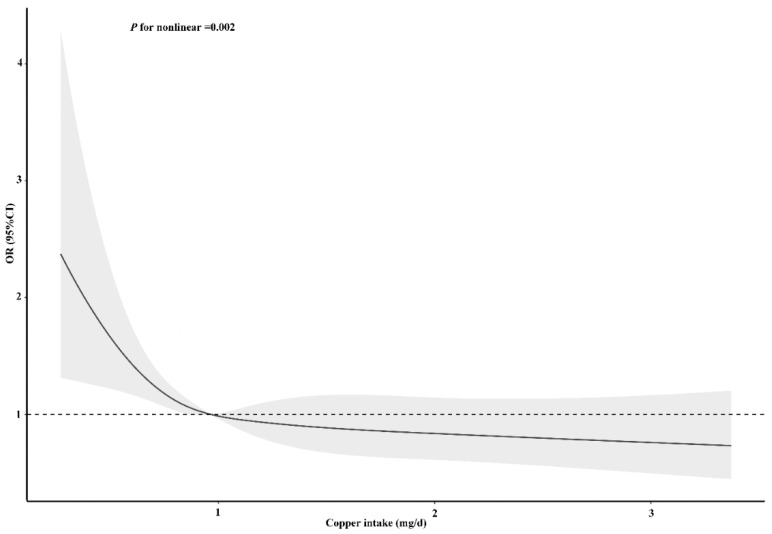
Restricted cubic spline models for the relationship between dietary copper intake and the risk of stroke after matching. The 95% CIs of the adjusted ORs are represented by the gray-shaded area. The model is adjusted for sex, age, race (Mexican American, other Hispanic, non-Hispanic white, non-Hispanic black, or other race), education (less than 9th grade, 9–11th grade, high school, graduate/GED or equivalent, some college or AA degree, or college graduate or above), PIR, marital status (married/living with partner, widow/divorced/separated, or never married), insurance, smoking, hypertension, HDL, asthma, arthritis, diabetes (yes, no, or borderline), general health (excellent, very good/good, or fair/poor), PHQ-9 depression score, trouble sleeping, and epilepsy. OR, odds ratio.

To clarify the relationship between dietary copper intake and stroke risk more clearly, we conducted an RCS analysis before and after matching. In both the unadjusted (Figure S1) and adjusted models (Figure 5), we found that there was an L-shaped association between dietary copper intake and stroke risk. The prevalence of stroke decreased with the increase of dietary copper intake and showed a non-linear relationship (*p* for nonlinear < 0.001). After matching, the results of the unadjusted (Figure S2) (*p* for nonlinear < 0.001) and adjusted (Figure 6) (*p* for nonlinear = 0.002) RCS models also showed an L-shaped nonlinear relationship between dietary copper intake and stroke risk.

### 3.4. Associations between Copper Intake and Baseline Data

**Figure 7:**
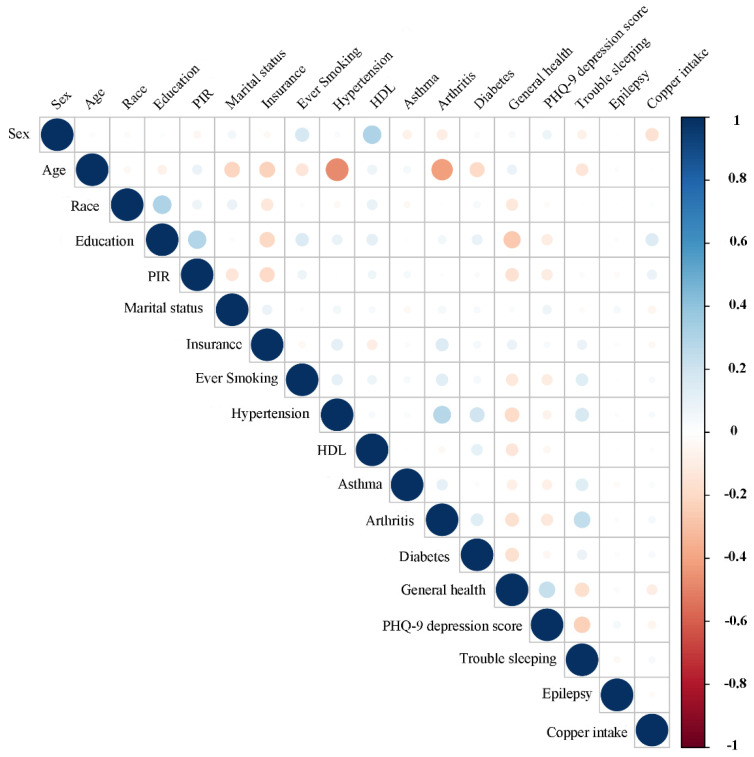
Correlation between the dietary copper intake and the baseline data in the control group. The color represents the association’s strength. The Pearson correlation coefficient was used to calculate the correlation.

**Figure 8:**
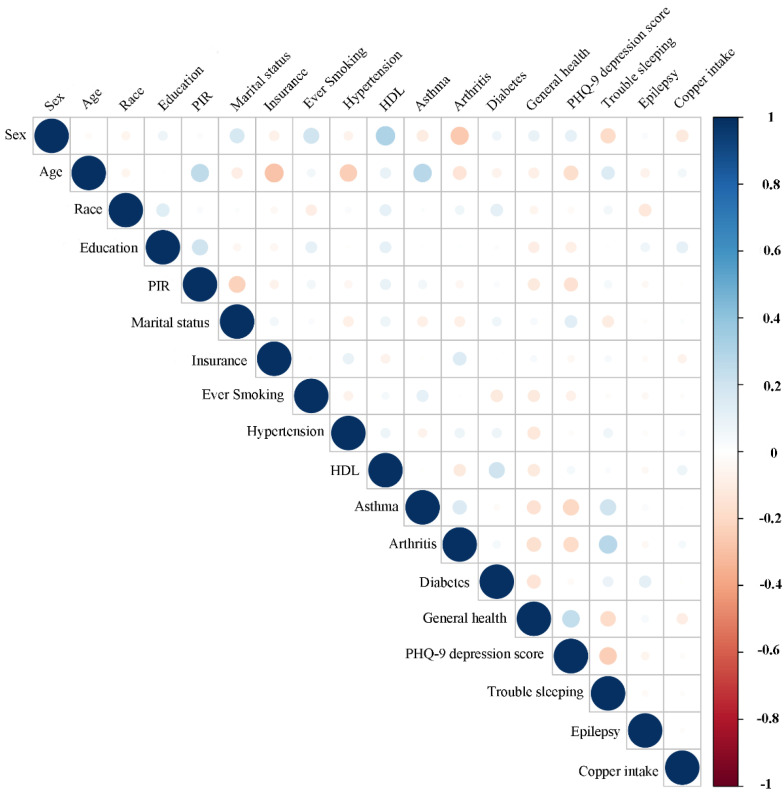
Correlation between the dietary copper intake and the baseline data in the stroke group. The color represents the association’s strength. The Pearson correlation coefficient was used to calculate the correlation.

For stroke and control subjects, the associations between dietary copper intake and baseline data were evaluated (Figure 7 and Figure 8). Our results showed that copper intake was positively correlated with education (r = 0.15, *p* < 0.001), PIR (r = 0.08, *p* <0.001), smoking (r = 0.032, *p* = 0.001), hypertension (r = 0.03, *p* = 0.002), asthma (r = 0.03, *p* = 0.006), arthritis (r = 0.04, *p* < 0.001), diabetes (r = 0.04, *p* < 0.001), and trouble sleeping (r = 0.04, *p* < 0.001) but negatively correlated with sex (r = −0.15, *p* < 0.001), marital status (r = −0.05, *p* < 0.001), insurance (r = −0.035, *p* < 0.001), general health (r = −0.09, *p* < 0.001), and PHQ-9 depression score (r = −0.06, *p* < 0.001) in the control group. However, copper intake was positively correlated with education (r = 0.1, *p* = 0.036) but negatively correlated with sex (r = −0.12, *p* = 0.019) in the stroke group.

## 4. Discussion

This large retrospective study explored the potential relationship between dietary copper intake and the risk of stroke by merging and analyzing NHANES data from 2013 to 2018. First of all, we also found that age, hypertension, and diabetes were risk factors for stroke. This was consistent with previous findings, and there are numerous pieces of evidence supporting the relationship between them. The most important finding of this study was that the risk of stroke decreased with the increase of dietary copper intake. In order to reduce case selection and clinical confounding factors bias, we conducted a PSM analysis. Combined with the results of the RCS analysis, we believe that dietary copper intake might be a protective factor for stroke. In addition, a further stratified analysis revealed that dietary copper intake was a significant protective factor for females, individuals <65 years old, individuals with hypertension, individuals who smoke, and diabetic stroke patients.

The relationship between copper and stroke risk has been reported in some studies. In a case-control study, Yang et al. believed that plasma copper was significantly related to the high risk of ischemic stroke, but not related to hemorrhagic stroke [12]. Another meta-analysis showed that the concentration of serum copper in the ischemic stroke group was significantly higher than that in the control group, suggesting that high serum copper concentration was a risk factor for ischemic stroke [30]. In addition, studies have shown that serum copper is not only positively correlated with the severity of atherosclerosis, but also significantly correlated with the increase of serum triglycerides [10,31]. However, there are some research reports that do not support the above views. Klevay et al. believed that copper could maintain the toughness and elasticity of blood vessel wall fibers as a component of superoxide dismutase and monoamine oxidase. Cholesterol degradation and plasma lipoprotein metabolism disorder caused by copper deficiency could deposit cholesterol on the damaged blood vessel wall, which ultimately leads to the formation of atherosclerosis [32]. A case-control study showed no association between serum copper and stroke risk (OR = 2.26, 95% confidence interval: 0.65–7.88). The Karadas study showed that the copper concentration of patients with acute hemorrhagic stroke was lower than that of the control group [13,33].

However, to our knowledge, studies on dietary copper intake and stroke risk are relatively limited, and our results found a negative correlation between dietary copper intake and stroke risk. These conflicting results may be due to the following: First of all, copper has two sides; it is both a pro-oxidant and an antioxidant. It is indispensable for a variety of enzymatic reactions related to metabolism and oxidative stress, such as ceruloplasmin and copper-dependent superoxide dismutase (SOD) [34,35]. A rabbit experiment showed that excessive or insufficient copper intake increased the susceptibility to atherosclerosis, which reflected the biphasic relationship between copper and atherosclerosis [36]. Second, the characteristics of each observational study, stroke subtype, specimen type, sample size, and other adjusted confounding factors are also different. In particular, there are few studies that systematically evaluate whether the correlation between copper and stroke varies according to the subtype and stage of stroke. In the acute stage of stroke, the stressed human body will release catecholamine synthase containing copper due to the excitation of the sympathetic nerve, which will increase the level of serum copper [30]. Finally, dietary copper intake does not represent blood copper levels, because blood copper levels vary with a variety of factors. Bost et al. showed that there was no change in plasma copper levels when the dietary copper intake was 0.57 to 6.0 mg/day [37]. Therefore, more large-scale clinical studies with clear subtypes and stages of stroke are needed in the future to clarify the relationship between dietary copper intake and the risk of stroke.

However, our research still has some limitations. Firstly, the dietary copper intake in this study was collected through two 24-h recalls, which might have recall bias and can not accurately reflect the individual’s usual copper intake. In addition, even for the same food, the content of copper may vary from place to place, so the assessment of copper intake by this method may not be accurate enough. However, some studies have shown that daily dietary intake may be sufficient to be evaluated based on two 24-h recalls [38]. Second, the inclusion criteria for stroke relied on self-reported stroke history, and the subtype and stage of stroke were not clear. Third, this study only analyzed copper intake without exploring the relationship between other dietary types and stroke incidents. Such as dietary fiber and fat, etc., these may play an important role in the occurrence and development of different subtypes of stroke. However, since NHANES data are not comprehensive enough, we will further analyze their relationship with different subtypes of stroke in future studies. In addition, some stroke subjects might have stroke sequelae of poor appetite or reduced eating ability, which affected copper intake. Finally, although we included many covariates in the analysis model, we still could not exclude the confounding effects of unincluded or unknown factors. Moreover, we were unable to assess the causal relationship between these factors because this was a cross-sectional study. Further prospective studies are needed to confirm these findings.

## 5. Conclusions

This is a large cross-sectional study to evaluate the association between dietary copper intake and the risk of stroke, based on the NAHNES database. We found that the risk of stroke decreased with increasing dietary copper intake. Moreover, dietary copper intake was a significant protective factor for women, individuals <65 years old, individuals with hypertension, individuals who smoke, and diabetic stroke patients. This may provide a basis for preventing stroke by adjusting dietary copper intake.

## Figures and Tables

**Table 1:** Baseline characteristics for the total subjects, as well as stratified by quartile of copper intake.

Characteristic	Total Subjects (N = 10,550)	Copper Intake Quartile, mg/day			
		Q1 (<0.799)	Q2 (≥0.799 to <1.072)	Q3 (≥1.072 to <1.42)	Q4 (≥1.42)
Sex (%)					
Male	5036 (47.7)	932 (35.4)	1156 (43.8)	1325 (50.3)	1623 (61.5)
Female	5514 (52.3)	1703 (64.6)	1483 (56.2)	1310 (49.7)	1018 (38.5)
Age (y), Mean (SD)	50 (17.35)	50.07 (17.99)	50.57 (17.8)	50.22 (17.11)	49.14 (16.43)
Race (%)					
Mexican American	1452 (13.8)	278 (10.6)	402 (15.2)	386 (14.6)	386 (14.6)
Other Hispanic	1040 (9.9)	279 (10.6)	250 (9.5)	250 (9.5)	261 (9.9)
Non-hispanic white	4374 (41.5)	1012 (38.4)	1144 (43.3)	1123 (42.6)	1095 (41.5)
Non-hispanic black	2183 (20.7)	809 (30.7)	538 (20.4)	490 (18.6)	346 (13.1)
Other race	1501 (14.2)	257 (9.8)	305 (11.6)	386 (14.6)	553 (20.9)
Education (%)					
Less than 9th grade	695 (6.6)	222 (8.4)	186 (7.0)	167 (6.3)	120 (4.5)
9–11th grade	1177 (11.2)	390 (14.8)	319 (12.1)	244 (9.3)	224 (8.5)
High school graduate/GED or equivalent	2441 (23.1)	777 (29.5)	652 (24.7)	554 (21)	458 (17.3)
Some college or AA degree	3425 (32.5)	880 (33.4)	863 (32.7)	867 (32.9)	815 (30.9)
College graduate or above	2812 (26.7)	366 (13.9)	619 (23.5)	803 (30.5)	1024 (38.8)
PIR (%)					
<1	2036 (19.3)	741 (28.1)	509 (19.3)	432 (16.4)	354 (13.4)
≥1	8514 (80.7)	1894 (71.9)	2130 (80.7)	2203 (83.6)	2287 (86.6)
Marital status (%)					
Married/living with partner	6399 (60.7)	1365 (51.8)	1575 (59.7)	1694 (64.3)	1765 (66.8)
Widow/divorced/separated	2293 (21.7)	717 (27.2)	621 (23.5)	520 (19.7)	435 (16.5)
Never married	1858 (17.6)	553 (21)	443 (16.8)	421 (16)	441 (16.7)
Insurance (%)					
Yes	8789 (83.3)	2146 (84.4)	2184 (82.8)	2192 (83.2)	2267 (85.8)
No	1761 (16.7)	489 (18.6)	455 (17.2)	443 (16.8)	374 (14.2)
Ever Smoking (%)					
Yes	4574 (43.4)	1261 (47.9)	1139 (43.2)	1109 (42.1)	1065 (40.3)
no	5976 (56.6)	1374 (52.1)	1500 (56.8)	1526 (57.9)	1576 (59.7)
Hypertension (%)					
Yes	4700 (44.5)	1257 (47.7)	1214 (46)	1152 (43.7)	1077 (40.8)
No	5850 (55.5)	1378 (52.3)	1425 (54)	1483 (56.3)	1564 (59.2)
HDL (mg/dL), Mean (SD)	53.36 (16.35)	53.43 (16.47)	53.30 (16.35)	53.51 (16.36)	53.21 (16.21)
Asthma (%)					
Yes	1650 (15.6)	514 (19.5)	404 (15.3)	361 (13.7)	371 (14)
No	8900 (84.4)	2121 (80.5)	2235 (84.7)	2274 (86.3)	2270 (86)
Arthritis (%)					
Yes	3050 (28.9)	858 (32.6)	761 (28.8)	764 (29)	667 (25.3)
No	7500 (71.1)	1777 (67.4)	1878 (71.2)	1871 (71)	1974 (74.7)
Stroke (%)					
Yes	399 (3.8)	155 (5.9)	99 (3.8)	83 (3.1)	62 (2.3)
No	10,151 (96.2)	2480 (94.1)	2540 (96.2)	2552 (96.9)	2579 (97.7)
Diabetes (%)					
Yes	1602 (15.2)	443 (16.8)	441 (16.7)	389 (14.8)	329 (12.5)
No	8646 (82)	2135 (81)	2121 (80.4)	2168 (82.3)	2222 (84.1)
Borderline	302 (2.9)	57 (2.2)	77 (2.9)	78 (3)	90 (3.4)
General health (%)					
Excellent	934 (8.9)	176 (6.7)	224 (8.5)	223 (8.5)	311 (11.8)
Very good/good	7121 (67.5)	1662 (63.1)	1760 (66.7)	1816 (68.9)	1883 (71.3)
Fair/poor	2495 (23.6)	797 (30.2)	655 (24.8)	596 (22.6)	447 (16.9)
PHQ-9 depression score (%)					
<10	9608 (91.1)	2297 (87.2)	2407 (91.2)	2434 (92.4)	2469 (93.5)
≥10	942 (8.9)	337 (12.8)	232 (8.8)	201 (7.6)	172 (6.5)
Trouble sleeping (%)					
Yes	3104 (29.4)	872 (33.1)	795 (30.1)	720 (27.3)	717 (27.1)
No	7446 (70.6)	1763 (66.9)	1844 (69.9)	1915 (72.7)	1924 (72.9)
Epilepsy (%)					
No	10,459 (99.1)	2603 (98.8)	2617 (99.2)	2612 (99.1)	2627 (99.5)
Yes	91 (0.9)	32 (1.2)	22 (0.8)	23 (0.9)	14 (0.5)

SD, standard deviation; PIR, poverty impact ratio; PHQ, patient health questionnaire; HDL, high-density lipoprotein.

**Table 2:** Characteristics of full and propensity score–matched cohorts by stroke.

	Before Matching			After Matching		
Variables	Control Group (*n* = 10,151)	Stroke Group (*n* = 399)	*p*	Control Group (*n* = 798)	Stroke Group (*n* = 399)	*p*
Sex (%)			0.509			0.967
Male	4852 (47.8)	184 (46.1)		3367 (46)	184 (46.1)	
Female	5299 (52.2)	215 (53.9)		431 (54)	215 (53.9)	
Age (y), Mean (SD)	49.41 (17.25)	65.01 (12.7)	<0.001	65.76 (11.79)	65.01 (12.7)	0.309
Race (%)			<0.001			0.048
Mexican American	1419 (14)	33 (8.3)		77 (9.6)	33 (8.3)	
Other Hispanic	1015 (10)	25 (6.3)		71 (8.9)	25 (6.3)	
Non-hispanic white	4178 (41.2)	196 (49.1)		352 (44.1)	196 (49.1)	
Non-hispanic black	2073 (20.4)	110 (27.6)		194 (24.3)	110 (27.6)	
Other race	1466 (14.4)	35 (8.8)		104 (13)	35 (8.8)	
Education (%)			<0.001			0.01
Less than 9th grade	655 (6.6)	30 (7.5)		95 (11.9)	30 (7.5)	
9-11th grade	1110 (10.9)	67 (16.8)		123 (15.4)	67 (16.8)	
High school graduate/GED or equivalent	2319 (22.8)	122 (30.6)		202 (25.3)	122 (30.6)	
Some college or AA degree	3302 (32.5)	123 (30.8)		221 (27.7)	123 (30.8)	
College graduate or above	2755 (27.1)	57 (14.3)		157 (19.7)	57 (14.3)	
PIR (%)			0.014			0.773
<1	1940 (19.1)	96 (24.1)		186 (23.3)	96 (24.1)	
≥1	8211 (80.9)	303 (75.9)		612 (76.7)	303 (75.9)	
Marital status (%)			<0.001			0.934
Married/living with partner	6178 (60.9)	221 (55.4)		433 (54.3)	221 (55.4)	
Widow/divorced/separated	2150 (21.2)	143 (35.8)		293 (36.7)	143 (35.8)	
Never married	1823 (18)	35 (8.8)		72 (9)	35 (8.8)	
Insurance (%)			<0.001			0.631
Yes	8420 (82.9)	369 (92.5)		744 (93.2)	369 (92.5)	
No	1731 (17.1)	30 (7.5)		54 (6.8)	30 (7.5)	
Ever Smoking (%)			<0.001			0.563
Yes	4341 (42.8)	233 (58.4)		452 (56.6)	233 (58.4)	
no	5810 (57.2)	166 (41.6)		346 (43.4)	166 (41.6)	
Hypertension (%)			<0.001			
Yes	4371 (43.1)	329 (82.5)		670 (84)	329 (82.5)	
No	5780 (56.9)	70 (17.5)		128 (16)	70 (17.5)	
HDL (mg/dL) Mean (SD)	53.38 (16.34)	52.82 (16.5)	0.503	53.14 (16.27)	52.82 (16.5)	0.751
Asthma (%)			<0.001			0.741
Yes	1548 (15.2)	102 (25.6)		197 (24.7)	102 (25.6)	
No	8603 (84.8)	297 (74.4)		601 (75.3)	297 (74.4)	
Arthritis (%)			<0.001			0.741
Yes	2818 (27.8)	232 (58.1)		456 (57.1)	232 (58.1)	
No	7333 (72.2)	167 (41.9)		342 (42.9)	167 (41.9)	
Diabetes (%)			<0.001			0.194
Yes	1451 (14.3)	151 (37.8)		277 (34.7)	151 (37.8)	
No	8417 (82.9)	229 (57.4)		495 (62)	229 (57.4)	
Borderline	283 (2.8)	19 (4.8)		26 (3.3)	19 (4.8)	
General health (%)			<0.001			0.473
Excellent	925 (9.1)	9 (2.3)		12 (1.5)	9 (2.3)	
Very good/good	6942 (68.4)	179 (44.9)		380 (47.6)	179 (44.9)	
Fair/poor	2284 (22.5)	211 (52.9)		406 (50.9)	211 (52.9)	
PHQ-9 depression score (%)			<0.001			0.504
<10	9283 (91.4)	325 (81.5)		637 (79.8)	325 (81.5)	
≥10	868 (8.6)	74 (18.5)		161 (20.2)	74 (18.5)	
Trouble Sleeping (%)			<0.001			0.682
Yes	2909 (28.7)	195 (48.9)		380 (47.6)	195 (48.9)	
No	7242 (71.3)	204 (51.1)		418 (52.4)	204 (51.1)	
Epilepsy (%)			<0.001			0.257
No	10080 (99.3)	379 (95)		769 (96.4)	379 (95)	
Yes	71 (0.7)	20 (5)		29 (3.6)	20 (5)	
Copper intake quartile (%)			<0.001			0.007
Q1	2480 (24.4)	155 (38.8)		178 (22.3)	121 (30.3)	
Q2	2540 (25)	99 (24.8)		195 (24.4)	104 (26.1)	
Q3	2552 (25.1)	83 (20.8)		211 (26.4)	88 (22.1)	
Q4	2579 (25.4)	62 (15.5)		214 (26.8)	86 (21.6)	

SD, standard deviation; PIR, poverty impact ratio; PHQ, patient health questionnaire; HDL, high-density lipoprotein.

## Data Availability

All NHANES data for this study are publicly available and can be found here: https://wwwn.cdc.gov/nchs/nhanes (accessed on 11 December 2021).

## Supplementary Materials

The following are available online at https://www.mdpi.com/article/10.3390/nu14030409/s1, Figure S1: Unadjusted restricted cubic spline models for the relationship between dietary copper intake and the risk of stroke before matching, Figure S2: Unadjusted restricted cubic spline models for the relationship between dietary copper intake and the risk of stroke after matching, Table S1: Association between copper intake and stroke as categorized by sex, Table S2: Association between copper intake and stroke as categorized by age, Table S3: Association between copper intake and stroke as categorized by hypertension, Table S4: Association between copper intake and stroke as categorized by smoking, Table S5: Association between copper intake and stroke as categorized by diabetes.

## References

[1] Mironczuk A., Kapica-Topczewska K., Socha K., Soroczynska J., Jamiolkowski J., Kulakowska A., Kochanowicz J. (2021). Selenium, Copper, Zinc Concentrations and Cu/Zn, Cu/Se Molar Ratios in the Serum of Patients with Acute Ischemic Stroke in Northeastern Poland-A New Insight into Stroke Pathophysiology. Nutrients.

[2] Mai X., Liang X. (2020). Risk Factors for Stroke Based on the National Health and Nutrition Examination Survey. J. Nutr. Health Aging.

[3] Huang S.W., Chi W.C., Chang K.H., Yen C.F., Liao H.F., Escorpizo R., Liou T.-H. (2017). World health organization disability assessment schedule 2.0 as an objective assessment tool for predicting return to work after a stroke. Disabil. Rehabil..

[4] Benjamin E.J., Virani S.S., Callaway C.W., Chamberlain A.M., Chang A.R., Cheng S., Chiuve S.E., Cushman M., Delling F.N., Deo R. (2018). Heart Disease and Stroke Statistics-2018 Update: A Report From the American Heart Association. Circulation.

[5] Xu J., Xu G., Fang J. (2021). Association Between Serum Copper and Stroke Risk Factors in Adults: Evidence from the National Health and Nutrition Examination Survey, 2011–2016. Biol. Trace Elem. Res..

[6] Ackerman C.M., Chang C.J. (2018). Copper signaling in the brain and beyond. J. Biol. Chem..

[7] Hu L., Bi C., Lin T., Liu L., Song Y., Wang P., Wang B., Fang C., Ma H., Huang X. (2021). Association between plasma copper levels and first stroke: A community-based nested case-control study. Nutr. Neurosci..

[8] Scheiber I.F., Mercer J.F., Dringen R. (2014). Metabolism and functions of copper in brain. Prog. Neurobiol..

[9] Witt B., Schaumloffel D., Schwerdtle T. (2020). Subcellular Localization of Copper-Cellular Bioimaging with Focus on Neurological Disorders. Int. J. Mol. Sci..

[10] Bagheri B., Akbari N., Tabiban S., Habibi V., Mokhberi V. (2015). Serum level of copper in patients with coronary artery disease. Niger. Med. J..

[11] Zhang J., Cao J., Zhang H., Jiang C., Lin T., Zhou Z., Song Y., Li Y., Liu C., Liu L. (2019). Plasma copper and the risk of first stroke in hypertensive patients: A nested case-control study. Am. J. Clin. Nutr..

[12] Xiao Y., Yuan Y., Liu Y., Yu Y., Jia N., Zhou L., Wang H., Huang S., Zhang Y., Yang H. (2019). Circulating Multiple Metals and Incident Stroke in Chinese Adults. Stroke.

[13] Karadas S., Sayın R., Aslan M., Gonullu H., Katı C., Dursun R., Duran L., Gonullu E., Demir H. (2014). Serum levels of trace elements and heavy metals in patients with acute hemorrhagic stroke. J. Membr. Biol..

[14] Jiang Y., Wang L.-P., Dong X.-H., Cai J., Jiang G.-J., Zhang C., Xie H.-H. (2015). Trace Amounts of Copper in Drinking Water Aggravate Cerebral Ischemic Injury via Impairing Endothelial Progenitor Cells in Mice. CNS Neurosci. Ther..

[15] Juul F., Parekh N., Martinez-Steele E., Monteiro C.A., Chang V.W. (2021). Ultra-processed food consumption among US adults from 2001 to 2018. Am. J. Clin. Nutr..

[16] Mei Z., Addo O.Y., Jefferds M.E., Sharma A.J., Flores-Ayala R.C., Brittenham G.M. (2021). Physiologically based serum ferritin thresholds for iron deficiency in children and non-pregnant women: A US National Health and Nutrition Examination Surveys (NHANES) serial cross-sectional study. Lancet Haematol..

[17] Li S., Sun W., Zhang D. (2019). Association of Zinc, Iron, Copper, and Selenium Intakes with Low Cognitive Performance in Older Adults: A Cross-Sectional Study from National Health and Nutrition Examination Survey (NHANES). J. Alzheimers Dis..

[18] Wang L., Li S., Sanika G.H.A., Zhao J., Zhang H., Zhao L., Wang W. (2021). Association between Serum 25-Hydroxyvitamin D Level and Stroke Risk: An Analysis Based on the National Health and Nutrition Examination Survey. Behav. Neurol..

[19] Al-Ibrahim A.A., Jackson R.T. (2019). Healthy eating index versus alternate healthy index in relation to diabetes status and health markers in U.S. adults: NHANES 2007-2010. Nutr. J..

[20] Kroenke K., Spitzer R.L., Williams J.B.W. (2001). The PHQ-9: Validity of a brief depression severity measure. J. Gen. Intern. Med..

[21] Yu B., Zhang X., Wang C., Sun M., Jin L., Liu X. (2020). Trends in depression among Adults in the United States, NHANES 2005-2016. J. Affect. Disord..

[22] Yang L., Wang Y., Chen X., Zhang C., Chen J., Cheng H., Zhang L. (2021). Risk Factors for Epilepsy: A National Cross-Sectional Study from National Health and Nutrition Examination Survey 2013 to 2018. Int. J. Gen. Med..

[23] Semenov Y.R., Herbosa C.M., Rogers A.T., Huang A., Kwatra S.G., Cohen B., Anadkat M.J., Silverberg J.I. (2021). Psoriasis and mortality in the United States: Data from the National Health and Nutrition Examination Survey. J. Am. Acad. Dermatol..

[24] Cho S.-M., Mehaffey J.H., Meyers S.L., Cantor R.S., Starling R.C., Kirklin J.K., Jacobs J.P., Kern J., Uchino K., Yarboro L.T. (2021). Cerebrovascular Events in Patients With Centrifugal-Flow Left Ventricular Assist Devices: Propensity Score-Matched Analysis From the Intermacs Registry. Circulation.

[25] Kaster T.S., Vigod S.N., Gomes T., Sutradhar R., Wijeysundera D.N., Blumberger D.M. (2021). Risk of serious medical events in patients with depression treated with electroconvulsive therapy: A propensity score-matched, retrospective cohort study. Lancet Psychiatry.

[26] Rosenbaum P.R., Rubin D.B. (1983). The Central Role of the Propensity Score in Observational Studies for Causal Effects. Biometrika.

[27] Desquilbet L., Mariotti F. (2010). Dose-response analyses using restricted cubic spline functions in public health research. Stat. Med..

[28] Lee D.H., Keum N., Hu F.B., Orav E.J., Rimm E.B., Willett W.C., Giovannucci E.L. (2018). Predicted lean body mass, fat mass, and all cause and cause specific mortality in men: Prospective US cohort study. BMJ.

[29] Dong X., Li S., Sun J., Li Y., Zhang D. (2020). Association of Coffee, Decaffeinated Coffee and Caffeine Intake from Coffee with Cognitive Performance in Older Adults: National Health and Nutrition Examination Survey (NHANES) 2011-2014. Nutrients.

[30] Zhang M., Li W., Wang Y., Wang T., Ma M., Tian C. (2019). Association Between the Change of Serum Copper and Ischemic Stroke: A Systematic Review and Meta-Analysis. J. Mol. Neurosci..

[31] Wells E.M., Navas-Acien A., Apelberg B.J., Herbstman J.B., Jarrett J.M., Lin Y.H., Verdon C.P., Ward C., Caldwell K.L., Hibbeln J.R. (2014). Association of selenium and copper with lipids in umbilical cord blood. J. Dev. Orig. Health Dis..

[32] Klevay L.M. (2000). Cardiovascular disease from copper deficiency—A history. J. Nutr..

[33] Reunanen A., Knekt P., Marniemi J., Maki J., Maatela J., Aromaa A. (1996). Serum calcium, magnesium, copper and zinc and risk of cardiovascular death. Eur. J. Clin. Nutr..

[34] Festa R.A., Thiele D.J. (2011). Copper: An essential metal in biology. Curr. Biol..

[35] Salonen J.T., Salonen R., Korpela H., Suntioinen S., Tuomilehto J. (1991). Serum copper and the risk of acute myocardial infarction: A prospective population study in men in eastern Finland. Am. J. Epidemiol..

[36] Lamb D.J., Avades T.Y., Ferns G.A. (2008). Biphasic modulation of atherosclerosis induced by graded dietary copper supplementation in the cholesterol-fed rabbit. Int. J. Exp. Pathol..

[37] Bost M., Houdart S., Oberli M., Kalonji E., Huneau J.F., Margaritis I. (2016). Dietary copper and human health: Current evidence and unresolved issues. J. Trace Elem. Med. Biol..

[38] Knuppel S., Norman K., Boeing H. (2019). Is a Single 24-hour Dietary Recall per Person Sufficient to Estimate the Population Distribution of Usual Dietary Intake?. J. Nutr..

