# Supplementary material for: Dietary Copper Intake and Risk of Stroke in Adults: A Case-Control Study Based on National Health and Nutrition Examination Survey 2013–2018
Source: Nutrients. 2022 Jan 18;14(3):409. doi: 10.3390/nu14030409 (PMC8839334; doi:10.3390/nu14030409)
Supplement: Supplementary file 1 [file nutrients-14-00409-s001.zip › Table S1.pdf]

Table S1. Association between copper intake and stroke as categorized by sex

Group	Female				Group	Male			
	Unadjusted OR (95% CI)	*P* value	Multi-adjusted OR (95% CI)[a]	*P* value		Unadjusted OR (95% CI)	*P* value	Multi-adjusted OR (95% CI)[a]	*P* value
Copper intake quartile, mg/day		<0.001		0.002	Copper intake quartile, mg/day		<0.001		0.08
Q1 (<0.739)	1.00 (Ref)		1.00 (Ref)		Q1 (<0.885)	1.00 (Ref)		1.00 (Ref)	
Q2 (≥0.739 to <0.984)	0.61 (0.43,0.87)	0.006	0.68 (0.47,0.999)	0.049	Q2 (≥0.885 to <1.169)	0.71 (0.49,1.02)	0.064	0.82 (0.56,1.22)	0.331
Q3 (≥0.984 to <1.293)	0.41 (0.28,0.61)	<0.001	0.46 (0.3,0.7)	<0.001	Q3 (≥1.169 to <1.564)	0.5 (0.33,0.75)	0.001	0.71 (0.46,1.09)	0.118
Q4 (⩾1.293)	0.42 (0.29,0.63)	<0.001	0.59 (0.37,0.91)	0.017	Q4 (⩾1.564)	0.31 (0.19,0.49)	<0.001	0.52 (0.32,0.87)	0.013

Abbreviations: CI, confidence interval; OR, odds ratio.

[a]Multivariable model is adjusted for Age, Race (Mexican American, Other Hispanic, Non-hispanic white, Non-hispanic black, Other race), Education (Less than 9th grade,9-11th grade, High school, graduate/GED or equivalent, Some college or AA degree, College graduate or above), PIR, Marital status(Married/living with partner, Widow/divorced/separated, Never married), Insurance, Ever Smoking Hypertension, HDL, Asthma, Arthritis, Diabetes (Yes, No, Borderline), General health (Excellent, Very good/good, Fair/poor), PHQ-9 depression score, Trouble sleeping, Epilepsy.
