# Supplementary material for: Dietary Copper Intake and Risk of Stroke in Adults: A Case-Control Study Based on National Health and Nutrition Examination Survey 2013–2018
Source: Nutrients. 2022 Jan 18;14(3):409. doi: 10.3390/nu14030409 (PMC8839334; doi:10.3390/nu14030409)
Supplement: Supplementary file 1 [file nutrients-14-00409-s001.zip › Table S2.pdf]

Table S2. Association between copper intake and stroke as categorized by age

Group	Age <65 years				Group	Age ≥ 65years			
	Unadjusted OR (95% CI)	*P* value	Multi-adjusted OR (95% CI)[a]	*P* value		Unadjusted OR (95% CI)	*P* value	Multi-adjusted OR (95% CI)[a]	*P* value
Copper intake quartile, mg/day		<0.001		0.013	Copper intake quartile, mg/day		0.003		0.179
Q1 (<0.804)	1.00 (Ref)		1.00 (Ref)		Q1 (<0.78)	1.00 (Ref)		1.00 (Ref)	
Q2 (≥0.804 to <1.086)	0.5 (0.33,0.74)	<0.001	0.61 (0.4,0.92)	0.019	Q2 (≥0.78 to <1.024)	0.74 (0.51,1.06)	0.097	0.79 (0.54,1.15)	0.22
Q3 (≥1.086 to <1.445)	0.51 (0.34,0.75)	0.001	0.63 (0.41,0.96)	0.032	Q3 (≥1.024 to <1.36)	0.59 (0.4,0.86)	0.006	0.67 (0.45,1.01)	0.672
Q4 (≥1.445)	0.32 (0.21,0.51)	<0.001	0.49 (0.3,0.81)	0.005	Q4 (≥1.36)	0.51 (0.34,0.75)	0.001	0.68 (0.44,1.04)	0.075

Abbreviations: CI, confidence interval; OR, odds ratio.

[a]Multivariable model is adjusted for Sex, Race (Mexican American, Other Hispanic, Non-hispanic white, Non-hispanic black, Other race), Education (Less than 9th grade,9-11th grade, High school, graduate/GED or equivalent, Some college or AA degree, College graduate or above), PIR, Marital status(Married/living with partner, Widow/divorced/separated, Never married), Insurance, Ever Smoking Hypertension, HDL, Asthma, Arthritis, Diabetes (Yes, No, Borderline), General health (Excellent, Very good/good, Fair/poor), PHQ-9 depression score, Trouble sleeping, Epilepsy.
