# Supplementary material for: Dietary Copper Intake and Risk of Stroke in Adults: A Case-Control Study Based on National Health and Nutrition Examination Survey 2013–2018
Source: Nutrients. 2022 Jan 18;14(3):409. doi: 10.3390/nu14030409 (PMC8839334; doi:10.3390/nu14030409)
Supplement: Supplementary file 1 [file nutrients-14-00409-s001.zip › Table S3.pdf]

Table S3. Association between copper intake and stroke as categorized by hypertension

Group	Hypertension status-No				Group	Hypertension -Yes			
	**Unadjusted OR (95% CI)**	***P* value**	**Multi-adjusted OR (95% CI)[a]**	***P* value**		**Unadjusted OR (95% CI)**	***P* value**	**Multi-adjusted OR (95% CI)[a]**	***P* value**
Copper intake quartile, mg/day		0.297		0.971	Copper intake quartile, mg/day		<0.001		0.002
Q1 (<0.814)	1.00 (Ref)		1.00 (Ref)		Q1 (<0.776)	1.00 (Ref)		1.00 (Ref)	
Q2 (≥0.814 to <1.094)	0.66 (0.35,1.25)	0.206	0.86 (0.43,1.69)	0.654	Q2 (≥0.776 to <1.044)	0.71 (0.53,0.94)	0.018	0.75 (0.55,1.02)	0.064
Q3 (≥1.094 to <1.459)	0.7 (0.38,1.32)	0.271	0.97 (0.49,1.93)	0.936	Q3 (≥1.044 to <1.378)	0.53 (0.39,0.72)	<0.001	0.58 (0.42,0.81)	0.001
Q4 (≥1.459)	0.54 (0.27,1.06)	0.073	0.99 (0.46,2.14)	0.985	Q4 (≥1.378)	0.41 (0.3,0.57)	<0.001	0.55 (0.39,0.79)	0.001

Abbreviations: CI, confidence interval; OR, odds ratio.

[a]Multivariable model is adjusted for Sex, Age, Race (Mexican American, Other Hispanic, Non-hispanic white, Non-hispanic black, Other race), Education (Less than 9th grade,9-11th grade, High school, graduate/GED or equivalent, Some college or AA degree, College graduate or above), PIR, Marital status(Married/living with partner, Widow/divorced/separated, Never married), Insurance, Ever Smoking, HDL, Asthma, Arthritis, Diabetes (Yes, No, Borderline), General health (Excellent, Very good/good, Fair/poor), PHQ-9 depression score, Trouble sleeping, Epilepsy.
