# Supplementary material for: Dietary Copper Intake and Risk of Stroke in Adults: A Case-Control Study Based on National Health and Nutrition Examination Survey 2013–2018
Source: Nutrients. 2022 Jan 18;14(3):409. doi: 10.3390/nu14030409 (PMC8839334; doi:10.3390/nu14030409)
Supplement: Supplementary file 1 [file nutrients-14-00409-s001.zip › Table S4.pdf]

Table S4. Association between copper intake and stroke as categorized by smoking

Group	Ever smoking -No				Group	Ever smoking -Yes			
	**Unadjusted OR (95% CI)**	***P* value**	**Multi-adjusted OR (95% CI)[a]**	***P* value**		**Unadjusted OR (95% CI)**	***P* value**	**Multi-adjusted OR (95% CI)[a]**	***P* value**
Copper intake quartile, mg/day		<0.001		0.211	Copper intake quartile, mg/day		<0.001		0.027
Q1 (<0.818)	1.00 (Ref)		1.00 (Ref)		Q1 (<0.766)	1.00 (Ref)		1.00 (Ref)	
Q2 (≥0.818 to <1.091)	0.7 (0.47,1.03)	0.072	0.77 (0.51,1.17)	0.22	Q2 (≥0.766 to <1.047)	0.75 (0.54,1.05)	0.091	0.75 (0.55,1.02)	0.206
Q3 (≥1.091 to <1.449)	0.56 (0.37,0.85)	0.006	0.69 (0.44,1.09)	0.11	Q3 (≥1.047 to <1.388)	0.5 (0.35,0.73)	<0.001	0.58 (0.42,0.81)	0.005
Q4 (≥1.449)	0.34 (0.2,0.55)	<0.001	0.6 (0.35,1.03)	0.063	Q4 (≥1.388)	0.47 (0.32,0.69)	<0.001	0.55 (0.39,0.79)	0.034

Abbreviations: CI, confidence interval; OR, odds ratio.

[a]Multivariable model is adjusted for Sex, Age, Race (Mexican American, Other Hispanic, Non-hispanic white, Non-hispanic black, Other race), Education (Less than 9th grade,9-11th grade, High school, graduate/GED or equivalent, Some college or AA degree, College graduate or above), PIR, Marital status(Married/living with partner, Widow/divorced/separated, Never married), Insurance, HDL, Asthma, Arthritis, Diabetes (Yes, No, Borderline), General health (Excellent, Very good/good, Fair/poor), PHQ-9 depression score, Trouble sleeping, Epilepsy.
