# Supplementary material for: Dietary Copper Intake and Risk of Stroke in Adults: A Case-Control Study Based on National Health and Nutrition Examination Survey 2013–2018
Source: Nutrients. 2022 Jan 18;14(3):409. doi: 10.3390/nu14030409 (PMC8839334; doi:10.3390/nu14030409)
Supplement: Supplementary file 1 [file nutrients-14-00409-s001.zip › Table S5.pdf]

Table S5. Association between copper intake and stroke as categorized by diabetes

Group	Diabetes status-No				Group	Diabetes status -Yes, Borderline			
	Unadjusted OR (95% CI)	*P* value	Multi-adjusted OR (95% CI)[a]	*P* value		Unadjusted OR (95% CI)	*P* value	Multi-adjusted OR (95% CI)[a]	*P* value
Copper intake quartile, mg/day		<0.001		0.116	Copper intake quartile, mg/day		<0.001		0.013
Q1 (<0.803)	1.00 (Ref)		1.00 (Ref)		Q1 (<0.78)	1.00 (Ref)		1.00 (Ref)	
Q2 (≥0.803 to <1.079)	0.58 (0.41,0.82)	0.002	0.71 (0.49,1.03)	0.069	Q2 (≥0.78 to <1.04)	0.61 (0.41,0.92)	0.019	0.65 (0.42,1.01)	0.057
Q3 (≥1.079 to <1.437)	0.52 (0.37,0.75)	<0.001	0.67 (0.46,0.98)	0.041	Q3 (≥1.04 to <1.37)	0.53 (0.35,0.81)	0..003	0.57 (0.36,0.9)	0.016
Q4 (≥1.437)	0.41 (0.28,0.6)	<0.001	0.7 (0.46,1.06)	0.094	Q4 (≥1.37)	0.35 (0.23,0.59)	<0.001	0.46 (0.27,0.77)	0.003

Abbreviations: CI, confidence interval; OR, odds ratio.

[a]Multivariable model is adjusted for Sex, Age, Race (Mexican American, Other Hispanic, Non-hispanic white, Non-hispanic black, Other race), Education (Less than 9th grade,9-11th grade, High school, graduate/GED or equivalent, Some college or AA degree, College graduate or above), PIR, Marital status(Married/living with partner, Widow/divorced/separated, Never married), Insurance, Ever Smoking Hypertension, HDL, Asthma, Arthritis, General health (Excellent, Very good/good, Fair/poor), PHQ-9 depression score, Trouble sleeping, Epilepsy.
