# Supplementary figures and images for: Dietary Copper Intake and Risk of Stroke in Adults: A Case-Control Study Based on National Health and Nutrition Examination Survey 2013–2018
Source: Nutrients. 2022 Jan 18;14(3):409. doi: 10.3390/nu14030409 (PMC8839334; doi:10.3390/nu14030409)

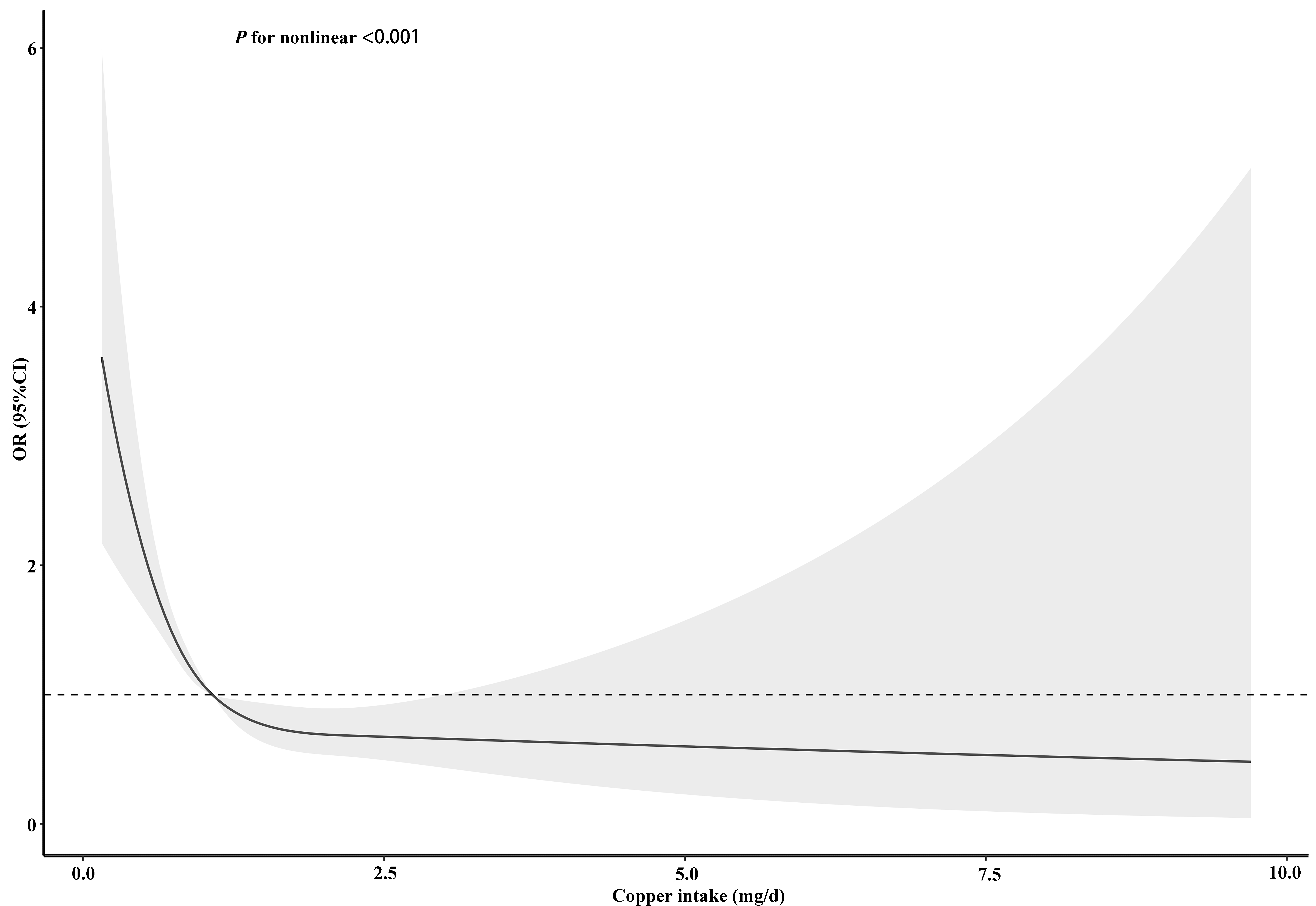

Supplement: Supplementary file 1 [file nutrients-14-00409-s001.zip › Figure S1.tif]

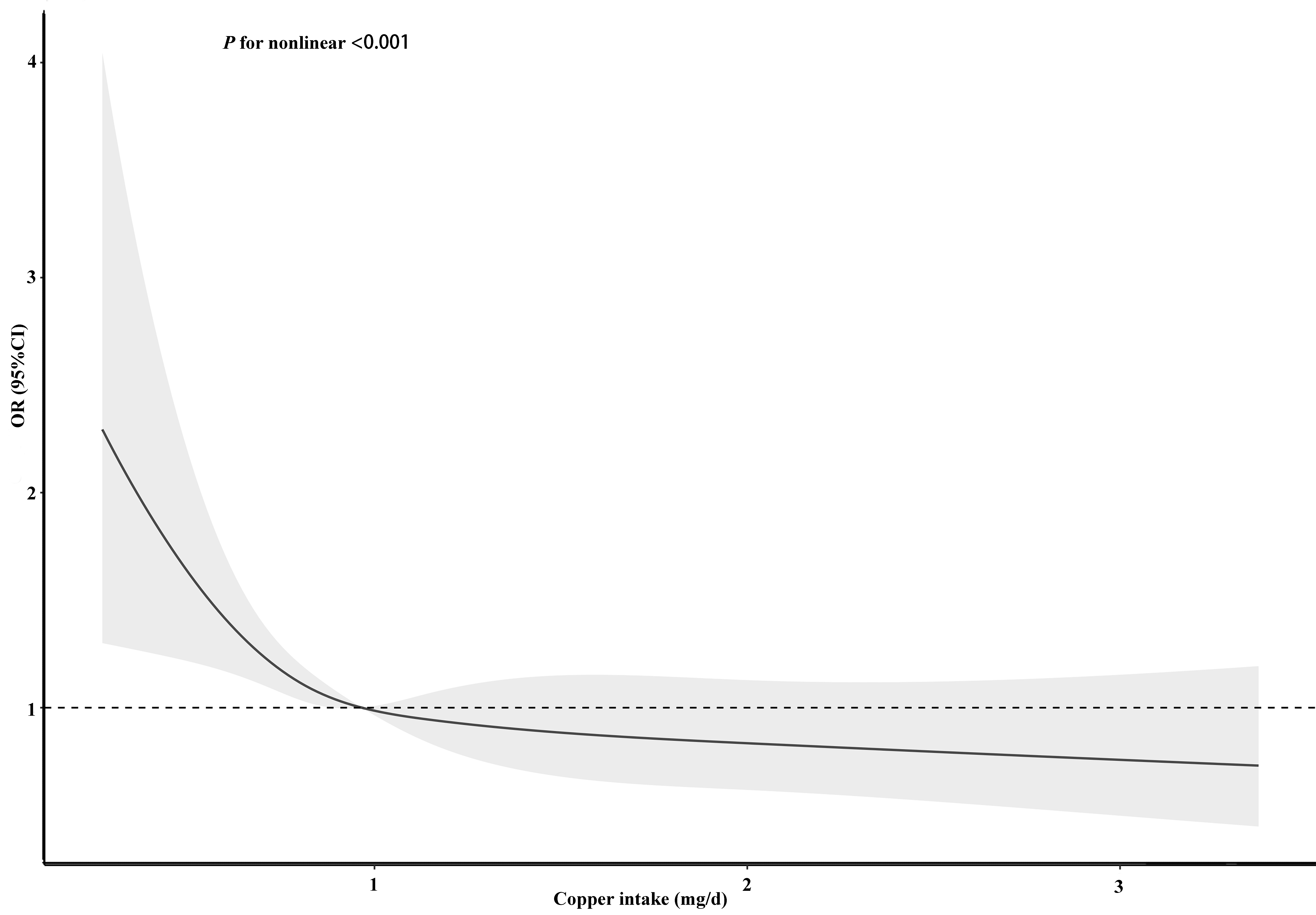

Supplement: Supplementary file 1 [file nutrients-14-00409-s001.zip › Figure S2.tif]
